# First-Principle Derivation of Single-Photon Entropy and Maxwell–Jüttner Velocity Distribution

**DOI:** 10.3390/e24111609

**Published:** 2022-11-04

**Authors:** Changhao Li, Jianfeng Li, Yuliang Yang

**Affiliations:** The State Key Laboratory of Molecular Engineering of Polymers, Department of Macromolecular Science, Fudan University, Shanghai 200433, China

**Keywords:** entropy of photon, Jüttner velocity distribution, absorption and emission

## Abstract

This work is devoted to deriving the entropy of a single photon in a beam of light from first principles. Based on the quantum processes of light–matter interaction, we find that, if the light is not in equilibrium, there are two different ways, depending on whether the photon is being added or being removed from the light, of defining the single-photon entropy of this light. However, when the light is in equilibrium at temperature *T*, the two definitions are equivalent and the photon entropy of this light is hν/T. From first principles, we also re-derive the Jüttner velocity distribution showing that, even without interatomic collisions, two-level atoms will relax to the state satisfying the Maxwell–Jüttner velocity distribution when they are moving in blackbody radiation fields.

## 1. Introduction

Light plays an important role in non-equilibrium thermodynamics not only in light-driving processes, but also in some basic processes such as atoms interacting with each other by exchanging photons. Therefore, proper and accurate evaluation of the entropy of a single photon in a light beam is crucial for the formulation of the light-related thermodynamics. Although the entropy of a beam of light with a certain frequency ν is well derived as $S\left(\nu \right)=k_{B}\left[\left(\langle n_{\nu }\rangle +1\right)\ln\left(\langle n_{\nu }\rangle +1\right)-\langle n_{\nu }\rangle \ln\langle n_{\nu }\rangle \right]$ with $\langle n_{\nu }\rangle$ the average number of photons [1], there still exist several different definitions of the entropy of a single photon in a beam of light. The first one is the average entropy, $s\left(\nu \right)=S\left(\nu \right)/\langle n_{\nu }\rangle$ [2], which is only a mathematical definition lacking proper physical realization [3]. The second one is the intrinsic entropy of a single photon [3,4,5], which is based on the observation that photons do not interact with each other and each of them thus forms an isolated thermodynamical system with the intrinsic entropy a constant independent of its frequency and the source temperature. The third definition cares about the effective entropy of a single photon, which is defined as the entropy change due to adding or removing a photon from the light [6,7,8,9]. Apart from the three definitions above, there still exists the fourth definition as $s\left(\nu ,T\right)=h\nu /[T\left(\exp h\nu /k_{B}T-1\right)]$ based on the classical definition of entropy change dS=δQ/T [10]. Coexistence of these definitions of single-photon entropy reflects the complexity involved in the thermodynamics of lights. In this work, we shall limit our attention only to the third definition, and the term “single-photon entropy”, in this work, will specially refer to effective single-photon entropy.

Previously, based on the following two equilibrium assumptions, (i) the incoming light is initially in equilibrium and (ii) photons in the light can quickly equilibrate again after adding or removing a photon from the light; it can be proven that the single-photon entropy of this light with the frequency ν at *T* is s(ν)=hν/T, with *h* the Planck constant, and this result has been applied in addressing various problems [8,9,11,12,13,14,15,16,17]. For example, it has been previously applied to evaluate the entropy of a laser beam [14,15,16] and the entropy production in the photosynthesis [17].

However, little work has been completed to derive the single-photon entropy from first principles without resorting to the above equilibrium assumptions [6,7], which will obviously restrict its wider applications. Accordingly, several problems regarding the single-photon entropy still exist. First, in what circumstances is the formula s(ν)=hν/T accurate or how long will it take for the light to equilibrate? Second, what is the single-photon entropy if the light is initially *not* in equilibrium? Third, even for an equilibrium light, can we derive, from first principles, the photon entropy of this light without assuming the light will quickly equilibrate again?

In Dirac’s work [18], he introduced the idea of second quantization and gracefully derived the Einstein coefficient relation from first principles without resorting to the equilibrium assumptions. Deeply inspired by Dirac’s work, this work closely follows the first principles to re-derive the single-photon entropy and address the above problems from first principles. We also extend this way of derivation to re-derive Jüttner velocity distribution [19] of two-level atoms in blackbody radiation fields. Apart from the derivation, the result itself is also an obvious example to demonstrate the importance of light in non-equilibrium thermodynamics and a connection between thermal motion and thermal radiation.

## 2. Single-Photon Entropy Evaluated by Examining the Light–Atom Interaction

Consider a beam of light with the frequency ν and the number distribution function of photons P(n) (or with the state specified by the density operator $\hat{\rho }=\sum_{n}P\left(n\right)|n\rangle \langle n|$). The total entropy of this light is obviously given by $S=-k_{B}\mathrm{Tr}\hat{\rho }\ln\hat{\rho }$ or $S=-k_{B}\sum_{n}P\left(n\right)\ln P\left(n\right)$. If the light is in equilibrium, then P(n) satisfies the Bose–Einstein distribution, i.e., P(n)=$e^{-nh\nu /k_{B}T}/\left(1-e^{-h\nu /k_{B}T}\right)$, and the temperature of this light can be determined by P(n) as $T=h\nu /k_{B}\ln P\left(n\right)/P\left(n+1\right)$. Here, the light is in equilibrium means that, if this beam of light interacts with atoms, the number density distribution P(n) of photons in the light is, on average, constant, and equating the absorption rate to the emission rate of photons leads to the equilibrium number distribution.

However, in the following derivation, we assume that P(n) can be any number distribution function to make the conclusion more general. Note that, even for a light that is not in equilibrium, it is possible to define its temperature according to recent works [20,21]. Furthermore, we assume that this beam of light is shining on some atoms *A* and some of the photons will be absorbed by *A*. After the time span Δt, the number distribution of the outgoing light will change a little due to the absorption. We emphasize that the following derivation does not assume that the light beam will equilibrate again after a photon of the beam has been absorbed by some atoms. Comparing the incoming and outgoing number distributions will lead to the entropy change of the beam.

Let us first compute the outgoing number distribution. For instance, at the time *t*, there are exactly *n* photons in the beam that are hitting the atom *A*. Then, there will be some probability of the state |A;n〉 turning into $|A^{*};n-1\rangle$ with $A^{*}$ the excited state of *A* and the energy gap between $A^{*}$ and *A* being $E_{A^{*}}-E_{A}=h\nu$. According to quantum field theory [18,22], the Hamiltonian of this process must look like $H=H_{I}^{†}a^{†}+H_{I}a$, where $H_{I}$ is an operator with non-zero matrix elements between different atom states, $H_{I}^{†}$ is its Hermitian conjugate and their precise expressions are not important here [18], and $a^{†}$ and *a* are the creation and annihilation operators of the photon, respectively. Then, during a small time span Δt, the amount of |A;n〉 that has been excited is proportional to $|\langle A^{*};n-{1|H|A;n\rangle |}^{2}\Delta t={\left|M_{0}\right|}^{2}n\Delta t=n\epsilon$ with $M_{0}=\langle A^{*}|H_{I}|A\rangle$, $\langle n-1|a^{†}|n\rangle =0$ and $\langle n-1|a|n\rangle =\sqrt{n}$ (see ref. [22]). Here, we define a new parameter ϵ ($\equiv |M_{0}{|}^{2}\Delta t$) for convenience, which is small for the small time interval Δt.

Therefore, the outgoing number distribution function becomes

(1) $$\begin{array}{c} P_{out}\left(n;\epsilon \right)=P\left(n+1\right)\cdot \left(n+1\right)\epsilon +P\left(n\right)\left(1-n\epsilon \right). \end{array}$$

where P(n+1)·(n+1)ϵ accounts for the probability of the occurrence of $\left|A;n+1\rangle \to \right|A^{*};n\rangle$ while P(n)(1−nϵ) accounts for the probability of |A;n〉 not being transformed into $|A^{*};n-1\rangle$.

Finally, the entropy of a single photon in a beam of light can be obtained by evaluating the entropy decrease per number of photons absorbed by *A* as

(2) $$\begin{array}{ccc} s_{ab}\left(\nu \right) & = & \lim_{\epsilon \to 0}\frac{\Delta S}{\Delta n}\\ & = & \lim_{\epsilon \to 0}\frac{-k_{B}\sum_{n=0}^{\infty }\left[P_{out}\left(n;\epsilon \right)\ln P_{out}\left(n;\epsilon \right)-P\left(n\right)\ln P\left(n\right)\right]}{-\sum_{n=0}^{\infty }n\epsilon P\left(n\right)}\\ & = & \frac{k_{B}}{\langle n_{\nu }\rangle }\sum_{n=0}^{\infty }\left(n+1\right)P\left(n+1\right)\ln\frac{P(n)}{P(n+1)}, \end{array}$$

where $\sum_{n=1}^{\infty }n\epsilon P\left(n\right)$ accounts for the number of photons absorbed by *A* and the averaged number of photons in the light is $\langle n_{\nu }\rangle =\sum_{n=1}^{\infty }nP\left(n\right)$. Thesubscript ab is used to emphasize that this single-photon entropy is defined by evaluating the entropy decrease per number of photons absorbed by *A*. Detailed derivation of Equation (2) is referred to Appendix A. Note that Equation (2) is valid for all kinds of light sources.

Similarly, the entropy of a single photon in a beam of light can be also obtained by evaluating the entropy increase per number of photons emitted by $A^{*}$ through $|A^{*};n\rangle \to |A;n+1\rangle$ (both spontaneous and stimulated emissions have been included in this single equation), as follows (see Appendix A):

(3) $$\begin{array}{ccc} s_{em}\left(\nu \right) & = & -\lim_{\epsilon \to 0}\frac{-k_{B}\sum_{n=0}^{\infty }\left[P_{out}\left(n\right)\ln P_{out}\left(n\right)-P\left(n\right)\ln P\left(n\right)\right]}{\sum_{n=0}^{\infty }\left(n+1\right)\epsilon P\left(n\right)}\\ & = & \frac{k_{B}}{\langle n_{\nu }\rangle +1}\sum_{n=0}^{\infty }\left(n+1\right)P\left(n\right)\ln\frac{P(n)}{P(n+1)} \end{array}$$

Therefore, for an arbitrary number distribution, there will be two different ways, depending on whether the photon is being added (Equation (3)) or being removed (Equation (2)) from the light, of defining the photon entropy, and these two definitions are usually not equivalent. When there are $\Delta n_{ab}$ photons being absorbed and $\Delta n_{em}$ photons being emitted by *A* and $A^{*}$, respectively, the entropy change can be evaluated as $\Delta S=-s_{ab}\left(\nu \right)\Delta n_{ab}+s_{em}\left(\nu \right)\Delta n_{em}$. Quite interestingly, the definition of the temperature of non-equilibrium quantum systems [20,21] is similar to that of the non-equilibrium light entropy. Just like the light has two possible definitions of entropy (Equations (2) and (3)) when the light is not in equilibrium, there are also two effective temperatures for the non-equilibrium light, depending on whether the heat is flowing towards the environment or is absorbed by the system, i.e., cool-down temperature $T_{c}$ and heat-up temperature $T_{h}$. In terms of P(n), $T_{c}=\min_{i\neq j}\frac{(i-j)h\nu }{k_{B}\ln P\left(i\right)/P\left(j\right)}$ and $T_{h}=\max_{i\neq j}\frac{(i-j)h\nu }{k_{B}\ln P\left(i\right)/P\left(j\right)}$ according to Lipka-Bartosik et al.’s work [21]. However, unfortunately, there are no clear relations between $T_{c/h}$ and $s_{ab/em}\left(\nu \right)$ as far as we are concerned.

If P(n)/P(n+1) is independent of *n*, then we can define the single-photon entropy as $s\left(\nu \right)=s_{ab}\left(\nu \right)=s_{em}\left(\nu \right)=k_{B}\ln\gamma \left(\nu \right)$ with γ(ν)=P(n)/P(n+1); and, similarly, $T_{c}=T_{h}$$=h\nu /k_{B}\ln\gamma \left(\nu \right)$. When the incoming light is in equilibrium, then $\gamma \left(\nu \right)=e^{h\nu /k_{B}T}$, and we have

(4) $$\begin{array}{c} s\left(\nu \right)=s_{ab}\left(\nu \right)=s_{em}\left(\nu \right)=\frac{h\nu }{T}, \end{array}$$

which agrees with previous works [6,7,8,9] and has been previously applied to evaluate the entropy of a monochromatic laser [16] and the entropy production in photosynthesis [17].

## 3. The Velocity Distribution of Two-Level Atoms in Blackbody Radiation Fields

Inspired by the first-principle derivation above, we find that we can derive the equilibrium velocity distribution of two-level atoms placed in blackbody radiation fields without referring to Boltzmann factor.

Assume that there are a number of two-level atoms which will not interact with each other moving in a blackbody radiation field, and the energy difference between *A* and the excited state $A^{*}$ is $h\nu_{0}$. Since the atoms are moving, the frequency of the photon absorbed/emitted may not be $\nu_{0}$ but will be altered by the Doppler effect. It is expressed as

(5) $$\nu =\frac{\nu_{0}\sqrt{1-\frac{v^{2}}{c^{2}}}}{1-\frac{\left|v\right|}{c}\cos\theta },$$

where θ is the angle between the directions of light and particle momentum.

At the same time, when the atoms absorb or emit photons, their momenta or velocities will change. Based on this kinetics, we can derive the equilibrium velocity distribution.

**Figure 1:**
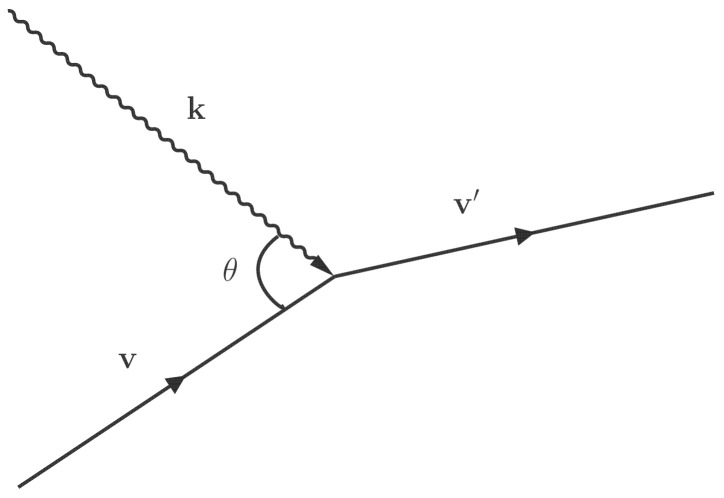
A demonstration of the absorption process.

For instance, consider an atom *A* with velocity v absorbing a photon with the wave vector k and changing into the excited state $A^{*}$ with velocity $v^{\prime }$ (see Figure 1), and assume that the Hamiltonian of this process can be written as $H=H_{I}^{†}a^{†}+H_{I}a$. Note that the velocity v, the wave vector k or the photon frequency ν=|k|c, and the frequency $\nu_{0}$ satisfy Equation (5). The probability for this absorption process is $p_{A}\left(v\right)|\langle A^{*},v^{\prime };n_{k}-1,k|H|A,v;n_{k}{,k\rangle |}^{2}=p_{A}\left(v\right)n_{k}|\langle A^{*},v^{\prime }|H_{I}{\left|A,v\rangle \right|}^{2}$. The excited state $A^{*}$ with velocity $v^{\prime }$ can also emit a photon with wave vector k (both spontaneous and stimulated emissions) and change back to atom *A* with velocity v. The probability for this emission process is $p_{A^{*}}\left(v^{\prime }\right)|\langle A,v;n_{k}+1,k|H|A^{*},v^{\prime };n_{k}{,k\rangle |}^{2}=p_{A^{*}}\left(v^{\prime }\right)\left(n_{k}+1\right)\left|\langle A,v\right|H_{I}^{†}|A^{*},v^{\prime }{\rangle |}^{2}=p_{A^{*}}\left(v^{\prime }\right)\left(n_{k}+1\right)|\langle A^{*},v^{\prime }|H_{I}{\left|A,v\rangle \right|}^{2}$.

When the system reaches equilibrium, the distribution will not change, which leads to $p_{A}\left(v\right)\langle n_{k}\rangle =p_{A^{*}}\left(v^{\prime }\right)\left(\langle n_{k}\rangle +1\right)$. Since the radiation field is blackbody-like, $\langle n_{k}\rangle ={\left[\exp\left(\beta h|k|c\right)-1\right]}^{-1}$ with $\beta =1/k_{B}T$ and *T* is the blackbody temperature. Therefore, we have

(6) $$p_{A^{*}}\left(v^{\prime }\right)=p_{A}\left(v\right)\exp\left(-\beta h|k|c\right).$$

Now, suppose that another atom with velocity v+dv can absorb a photon with the wave vector $k^{\prime }$ and change into the same excited state $A^{*}$ with the velocity $v^{\prime }$. Similarly, we have $p_{A^{*}}\left(v^{\prime }\right)=p_{A}\left(v+dv\right)\exp\left(-h|k^{\prime }|c/k_{B}T\right)$ in equilibrium and, comparing it with Equation (6), we obtain

(7) $$\frac{p_{A}\left(v+dv\right)}{p_{A}\left(v\right)}=\exp\left[-\beta h(|k|-|k^{\prime }|)c\right].$$

According to the energy conservation, the following relation must hold:

(8) $$\frac{mc^{2}}{\sqrt{1-{\left(\frac{v}{c}\right)}^{2}}}+h|k|c=\frac{mc^{2}}{\sqrt{1-{\left(\frac{v+dv}{c}\right)}^{2}}}+h\left|k^{\prime }\right|c,$$

where the left-hand side accounts for the total energy of the state |A,v〉 and a photon with k while the right-hand side is the total energy of the state |A,v+dv〉 and a photon with $k^{\prime }$. Substituting Equation (8) into Equation (7) and letting dv→0, we can obtain the equation that the velocity distribution satisfies,

(9) $$\begin{array}{c} \frac{d}{dv}p_{A}\left(v\right)=-\beta p_{A}\left(v\right)\frac{d}{dv}(\frac{mc^{2}}{\sqrt{1-{\left(\frac{v}{c}\right)}^{2}}}). \end{array}$$

More details about this equation: the left side of Equation (7) gives $1+\frac{dp_{A}\left(v\right)}{p_{A}\left(v\right)dv}$ and the right-hand side of Equation (7) gives $\exp\left\{\beta \left[\frac{mc^{2}}{\sqrt{1-{\left(\frac{v}{c}\right)}^{2}}}-\frac{mc^{2}}{\sqrt{1-{\left(\frac{v+dv}{c}\right)}^{2}}}\right]\right\}\approx \exp\left\{-dv\cdot \beta d\frac{mc^{2}}{\sqrt{1-{\left(\frac{v}{c}\right)}^{2}}}/dv\right\}$≈1−dv·βd$\frac{mc^{2}}{\sqrt{1-{\left(\frac{v}{c}\right)}^{2}}}/dv$ after Equation (8) has been plugged into this equation; equating these two expressions leads to the above equation. Noting that, in this section, we assume that all functions of v are isotropic in v and can be expressed in terms of |v|, then the operator d/dv in the above equation can be defined as $\frac{df(|v|)}{dv}\equiv \frac{df(|v|)}{d|v|}\frac{v}{\left|v\right|}$ for some function *f*.

Finally, the velocity distribution can be obtained, by integrating the above equation, as

(10) $$\begin{array}{c} p_{A}\left(v\right)\propto \exp\left(-\frac{\beta mc^{2}}{\sqrt{1-{\left(\frac{v}{c}\right)}^{2}}}\right). \end{array}$$

This velocity distribution is identical to the Jüttner distribution [19,23], which is reduced to the Maxwell velocity distribution for the small v. Note that the derivation above is unique and from first principles since we do not need to introduce the Boltzmann factor [24].

Finally, several comments are made.

**Figure 2:**
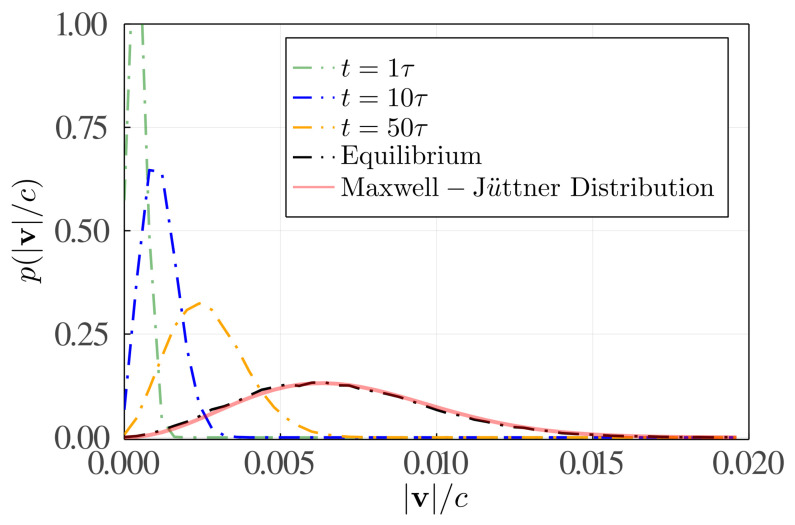
Radial distribution functions (dash-dotted lines) of 50,000 atoms’ velocities at different Monte Carlo (MC) simulation steps. The pale red solid line shows the Jüttner distribution at the same temperature. In this MC simulation, all atoms with the same velocity |v|=0.00017c but with different speed directions are placed in a blackbody radiation field, and interatomic collisions have not been considered. Parameter setting of the simulation is as follows: $h=c=k_{B}=1$, $\nu_{0}=3$, T=2, m=0.1 and a MC step Δt=0.001τ. For simplicity, it is assumed that $|\langle A^{*},v^{\prime }|H_{I}{\left|A,v\rangle \right|}^{2}\Delta t/\tau =0.5$ does not depend on atom’s velocities. *c* is the speed of light.

(i) In the original derivation [19] of Jüttner distribution, quantum effects were not considered. However, this work shows that, even when quantum effects are explicitly considered, through the second-quantization formulation, the atoms will still satisfy the distribution in the blackbody radiation field.

(ii) Apart from this unique way of derivation, the result itself is also interesting. Since these two-level atoms have no other internal energy level and no collisions (interactions) with each other, it is hard to believe that the velocity distribution can still relax to Maxwell–Jüttner form only by a blackbody radiation field because of the Doppler effect. Thus, we think this may be a new perspective to understand the relation between thermal motion and thermal radiation.

(iii) Even though we have proved, in theory, that atoms in a blackbody radiation field will relax to the Jüttner distribution even without interatomic collisions, it is still hard to imagine how this occurs. Therefore, we have performed a Monte Carlo (MC) simulation showing that the velocity distribution function of 50,000 atoms with the same initial absolute velocity (|v|=0.00017c) will gradually evolve to the Jüttner distribution (see Figure 2) without considering interatomic interactions. During each MC simulation step, we randomly select an atom and select all the model variables, such as k and θ, according to the probability or distributions given by the theory. According to Equation (5), an atom with the speed v has the possibility to absorb photons with the frequency ranging from $\nu_{0}\sqrt{1-\frac{v^{2}}{c^{2}}}/\left(1+\frac{\left|v\right|}{c}\right)$ to $\nu_{0}\sqrt{1-\frac{v^{2}}{c^{2}}}/\left(1-\frac{\left|v\right|}{c}\right)$ thanks to the Doppler effect, which might be one of the key reasons that the distribution widening without interatomic collisions is possible.

(iv) Note that Equation (4) in the last section can be also applied to estimate the entropy change of photons due to the absorption and emission processes discussed here, and it is $\Delta s_{\nu }\propto \left[P_{A^{*}}\left(v^{\prime }\right)\left(\langle n_{k}\rangle +1\right)-P_{A}\left(v\right)\langle n_{k}\rangle \right]\langle A^{*},v^{\prime }|H_{I}{\left|A,v\rangle \right|}^{2}h\left|k\right|c/T$ with *T* the temperature of the blackbody.

## 4. Conclusions

We have derived the entropy of a single photon from first principles and properly addressed the three problems proposed in the Introduction as follows: (i) The single-photon entropy s=hν/T is accurate as long as the light is initially in equilibrium, since our first-principle derivation shows that the outgoing light does not need to be in equilibrium again. (ii) If the light is initially not in equilibrium, then the single-photon entropy will be different from hν/T, and there will be two different ways of defining the single-photon entropy (Equations (2) and (3)). To our best knowledge, this result has not been reported before. (iii) We have successfully derived the single-photon entropy from first principles without assuming that the light will quickly equilibrate again.

From first principles without considering interatomic collisions, we have also derived the Jüttner velocity distribution of two-level atoms in the blackbody radiation field. The way of derivations and the results of both single-photon entropy and Jüttner distribution may provide a new perspective to understand the thermodynamical properties of lights as well as the connection between thermal motion and thermal radiation.

## Data Availability

Not applicable.

## Appendix A

First, it is easy to show that, if a function satisfies f(n)→0 for n→∞ and f(0)=0, then $\sum_{n=0}^{\infty }\left[f\left(n+1\right)-f\left(n\right)\right]=f\left(0\right)=0$. Notice that, from Equation (1), we have $P_{out}\left(n;\epsilon \right)=P\left(n+1\right)\cdot \left(n+1\right)\epsilon +P\left(n\right)\left(1-n\epsilon \right)=P\left(n\right)+\alpha \left(n\right)\epsilon$ with α(n)≡(n+1)P(n+1)−nP(n). Obviously, $\sum_{n=0}^{\infty }\alpha \left(n\right)=0$.

Therefore, we have

(A1) $$\begin{array}{c} \begin{array}{ccc} s_{ab}\left(\nu \right) & = & \lim_{\epsilon \to 0}\frac{k_{B}}{\sum_{n=0}^{\infty }n\epsilon P\left(n\right)}\sum_{n=0}^{\infty }\left[P_{out}\left(n;\epsilon \right)\ln P_{out}\left(n;\epsilon \right)-P\left(n\right)\ln P\left(n\right)\right]\\ & = & \lim_{\epsilon \to 0}\frac{k_{B}}{\langle n_{\nu }\rangle \epsilon }\sum_{n=0}^{\infty }\left\{\left[P\left(n\right)+\alpha \left(n\right)\epsilon \right]\ln P\left(n\right)\left[1+\frac{\alpha (n)}{P(n)}\epsilon \right]-P\left(n\right)\ln P\left(n\right)\right\}\\ & \dot{=} & \lim_{\epsilon \to 0}\frac{k_{B}}{\langle n_{\nu }\rangle \epsilon }\sum_{n=0}^{\infty }\left[\alpha \left(n\right)\epsilon \ln P\left(n\right)+\alpha \left(n\right)\epsilon \right]\\ & = & \frac{k_{B}}{\langle n_{\nu }\rangle }\sum_{n=0}^{\infty }\left[\alpha \left(n\right)\ln P\left(n\right)\right]\\ & = & \frac{k_{B}}{\langle n_{\nu }\rangle }\sum_{n=0}^{\infty }\left[\left(n+1\right)P\left(n+1\right)-nP\left(n\right)\right]\ln P\left(n\right)\\ & = & \frac{k_{B}}{\langle n_{\nu }\rangle }\sum_{n=0}^{\infty }[\left(n+1\right)P\left(n+1\right)\ln P\left(n+1\right)-nP\left(n\right)\ln P\left(n\right)\\ & & +(n+1)P(n+1)\ln P(n)-(n+1)P(n+1)\ln P(n+1)]\\ & = & \frac{k_{B}}{\langle n_{\nu }\rangle }\sum_{n=0}^{\infty }\left(n+1\right)P\left(n+1\right)\ln\frac{P(n)}{P(n+1)} \end{array} \end{array}$$

By applying the relation [P(n)+α(n)ϵ]ln[1+α(n)/ϵP(n)]≈[P(n)+α(n)ϵ]α(n)ϵ, where $\ln\left(1+x\right)\approx x-x^{2}/2$ for x→0 has been used, one obtains the third line from the second line. Note that, in the third line, the identity $\sum_{n=0}^{\infty }\alpha \left(n\right)=0$ has been applied and, in the last third line, $\sum_{n=0}^{\infty }\left[\left(n+1\right)P\left(n+1\right)\ln P\left(n+1\right)-nP\left(n\right)\ln P\left(n\right)\right]=0$ because of the above-mentioned identity $\sum_{n=0}^{\infty }\left[f\left(n+1\right)-f\left(n\right)\right]=0$ with f(n)=nP(n)lnP(n) here.Maybe nP(n)lnP(n)→0 as n→∞ is not so obvious. It can be proved as follows that ∑nP(n)=〈n〉 must be finite indicates at least nP(n)∼1/N for n→∞, where $N=\delta^{-1}$ with δ is a first-order infinitesimal number, which further leads to $P\left(n\right)∼1/N^{2}$ and lnP(n)∼−2lnN∼−lnN. Therefore, nP(n)lnP(n)∼−lnN/N→0 as n→∞ and N→∞.

The emission process (including spontaneous and stimulated ones) can be described by one process $|A^{*};n\rangle \to |A;n+1\rangle$ in quantum field theory. Similar to that of the absorption case, the transition rate of this process is proportional to n+1 since $\left|\langle n+1\right|a^{†}{\left|n\rangle \right|}^{2}=n+1$ with $a^{†}$ the creation operator. In addition, note that, for the emission case, the corresponding outgoing number distribution function becomes

(A2) $$\begin{array}{c} P_{out}\left(n;\epsilon \right)=P\left(n-1\right)\cdot n\epsilon +P\left(n\right)\left(1-\left(n+1\right)\epsilon \right)=P\left(n\right)+\alpha \left(n\right)\epsilon \end{array}$$

with $\epsilon =\left|\langle A\right|H_{I}|A^{*}{\rangle |}^{2}\Delta t$ and α(n)=P(n−1)·n−P(n)·(n+1) (note that α(0)=−P(0)). Therefore, similar to the derivation in Equation (A1), we have

(A3) $$\begin{array}{c} \begin{array}{ccc} s_{em}\left(\nu \right) & = & -\lim_{\epsilon \to 0}\frac{k_{B}}{\sum_{n=0}^{\infty }\left(n+1\right)\epsilon P\left(n\right)}\sum_{n=0}^{\infty }\left[P_{out}\left(n\right)\ln P_{out}\left(n\right)-P\left(n\right)\ln P\left(n\right)\right]\\ & = & -\frac{k_{B}}{\langle n_{\nu }\rangle +1}\sum_{n=0}^{\infty }\alpha \left(n\right)\ln P\left(n\right)\\ & = & -\frac{k_{B}}{\langle n_{\nu }\rangle +1}\sum_{n=0}^{\infty }\left[P\left(n-1\right)\cdot n-P\left(n\right)\cdot \left(n+1\right)\right]\ln P\left(n\right)\\ & = & -\frac{k_{B}}{\langle n_{\nu }\rangle +1}\sum_{n=0}^{\infty }[P\left(n-1\right)\cdot n\ln P\left(n\right)-P\left(n\right)\cdot \left(n+1\right)\ln P\left(n+1\right)\\ & & +P\left(n\right)\cdot \left(n+1\right)\ln\frac{P(n+1)}{P(n)}]\\ & = & \frac{k_{B}}{\langle n_{\nu }\rangle +1}\sum_{n=0}^{\infty }\left(n+1\right)P\left(n\right)\ln\frac{P(n)}{P(n+1)}. \end{array} \end{array}$$
